# Biogas Potential of the Side Streams Obtained in a Novel Phenolic Extraction System from Olive Mill Solid Waste

**DOI:** 10.3390/molecules25225438

**Published:** 2020-11-20

**Authors:** África Fernández-Prior, Ángeles Trujillo-Reyes, Antonio Serrano, Guillermo Rodríguez-Gutiérrez, Claudio Reinhard, Fernando G. Fermoso

**Affiliations:** 1 Instituto de Grasa, Spanish National Research Council (CSIC), Ctra. de Utrera, km. 1, 41013 Seville, Spain; mafprior@ig.csic.es (Á.F.-P.); atrujillo@ig.csic.es (Á.T.-R.); antonio.serrano@ig.csic.es (A.S.); guirogu@ig.csic.es (G.R.-G.); 2 School of Civil Engineering, The University of Queensland, Campus St. Lucia—AEB Ed 49, St. Lucia, QLD 4067, Australia; 3 Laboratory of Food Biochemistry, ETH Zurich, Schmelzbergstrasse 9, 8092 Zurich, Switzerland; claudio.reinhard@hest.ethz.ch

**Keywords:** mesophilic anaerobic digestion, valorisation, phenols, olive mill solid waste

## Abstract

The olive oil production is an important industrial sector in many Mediterranean areas, but it is currently struggled by the necessity of a proper valorisation of the olive mill solid waste or alperujo. The alperujo is the main by-product generated during the two-phase olive oil extraction, accounting for up to 80% of the initial olive mass. The alperujo is a source of valuable compounds, such as the pomace olive oil or highly interesting phenolic compounds. In the present research, a novel biorefinery approach has been used for phenolic compounds recovery. However, the extraction of these valuables compounds generates different exhausted phases with high organic matter content that are required to be managed. This study consists of the evaluation of the anaerobic biodegradability of the different fractions obtained in a novel biorefinery approach for the integral valorisation of alperujo. The results show that the different phases obtained during the biorefinery of the alperujo can be effectively subjected to anaerobic digestion and no inhibition processes were detected. The highest methane yield coefficients were obtained for the phases obtained after a two-months storages, i.e., suspended solids and liquid phase free of suspended solids, which generated 366 ± 7 mL CH_4_/g VS and 358 ± 6 mL CH_4_/g VS, respectively. The phenol extraction process reduced the methane yield coefficient around 25% due to the retention of biodegradable compounds during the extraction process. Regardless of this drop, the anaerobic digestion is a suitable technology for the stabilization of the different generated residual phases, whereas the high market price of the extracted phenols can largely compensate the slight decrease in the methane generation.

## 1. Introduction

Olive oil production is responsible for large amounts of agricultural by-products generally considered as waste to be disposed of. Only 20% of the olive fruit result in Extra-Virgin Oil [1]. Most Mediterranean countries face serious environmental and economic problems handling the waste produced in olive mills as its chemical composition creates challenges for stability, recycling and further processing. However, olive mill solid waste has some unique properties, most of all its high content in olive antioxidants, which makes this agricultural by-product a very interesting, abundant and low-cost resource for a wide range of value-added products [2].

Nowadays, the most common practice for dealing with olive mill solid waste is its storage in large evaporation ponds in order to reduce its water content by natural evaporation, resulting in a thickened sludge which is either completely dried and chemically extracted (pomace oil production) or directly disposed of in landfills. This practice causes several problems such as intensive land use, bad odour, GHG emission, soil and water contamination due to leakages, insect proliferation and a high energy demand for drying the pomace in pomace oil extraction plants [3]. Most of all, current practices do not make use of the valuable properties of olive mill solid waste such as its high content in antioxidants.

The sequential extraction of valuable compounds and/or energy from a by-product is called biorefinery [2]. The recovery of high value-added compounds would allow considering the olive mill solid waste as a source of new benefits, instead of a waste to be treated. A biorefinery system aims at combining different processes in a synergistic way, avoiding the implementation of processes that could compromise the subsequent processes. For example, different authors have reported that the application of high-temperature treatments for solubilizing the organic matter can generate high concentrations of furans and phenolic compounds, which would inhibit subsequent microbial-based processes [4]. The main phenolic compound to be recovered from the olive mill solid waste is the hydroxytyrosol (HT) [5], although the process could entail the increase in the concentration of 5-hydroxymethylfurfural, a well-defined inhibitory furan [4]. The extraction of the furans and phenolic compounds from the olive mill solid waste would then have a double benefit, i.e., the recovery of a fraction enriched in phenols and the detoxification of the remaining organic matter [6,7].

Anaerobic digestion is a valorisation method that has been widely proposed for the stabilization of biodegradable substrates, which might be an interesting option to recover energy and to stabilize the organic matter from the different fractions obtained from the biorefinery of the olive mill solid waste [2]. The extraction of the phenolic compounds solubilised during a thermal treatment would be beneficial for the subsequent anaerobic digestion process due to the inhibitory effect of these compounds over the anaerobic microorganisms, especially the methanogens [8]. However, the different extraction processes would limit the organic matter susceptible to be converted into methane. This limitation would result in the generation of no-biodegradable fractions.

In the present study, the biogas potential of the different side streams of a biorefinery approach for olive mill solid waste has been studied and evaluated. The main novelty presented is a feasibility study of a new phenolic extraction system by evaluating valorisation of the formed by-products by the application of a biological process such as anaerobic digestion. This would allow applying a biorefinery approach that is easily scalable to the industry level offering high value-added products, energy and digested substrates for use in agriculture. All these products replace or reduce the use of synthetic additives, chemical fertilizers and herbicides, so their use would also imply a very important environmental benefit.

## 2. Results and Discussion

### 2.1. Characterisation of the Side Streams Obtained

Table 1 summarizes the analytical characterization of the different phases used in the present study. These phases are a solid phase (SP), a liquid phase (LP), a suspended solids phase (SS) and a liquid phase free of suspended solids (LFP) as further explained in materials and methods section. As expected, SP presented the highest concentration of total solids (TS), i.e., 436,520 ± 7565 mg/kg, being the TS of the used alperujo before the three phase decanter 266,215 ± 7508 mg/kg. The TS concentration of the SP was around 21% higher than the TS concentration of other solid phases produced after centrifugation of alperujo in a two phase decanter [6,7]. The characterisation of the substrates indicated that most of the total solids corresponds to organic matter, reaching a VS/TS ratio higher than 74% in all the cases. It is worth to note that the liquid phases, i.e., LPF and DLP, presented a lower volatile solid (VS)/TS ratio than the solid phases, i.e., SP and SS, which reached values higher than 90%. This fact would mean that most of the mineral solids (MS) would correspond to dissolved salts such as carbonates or cations such as potassium, which is an important element in the alperujo [9].

The characterisation of the different phases showed a strong influence of the phenolic extraction process in the composition of the liquid phases (Table 1). The extraction process was highly effective for recovering the phenols, reducing up to 92% the total phenol concentrations, respectively, from LPF to DLP (Table 1). Additionally, the proposed extraction process also resulted in the reduction of the 88.9%, 90.3% and 96.8% of VS, sCOD and total sugars, respectively. This reduction in the sCOD would explain the increment in the pH from 4.9 ± 0.1 to 7.3 ± 0.1 during the phenolic extraction process.

### 2.2. Biogas Potential of the Side Streams Obtained

#### 2.2.1. Biogas Potential and Kinetic Study

**Figure 1:**
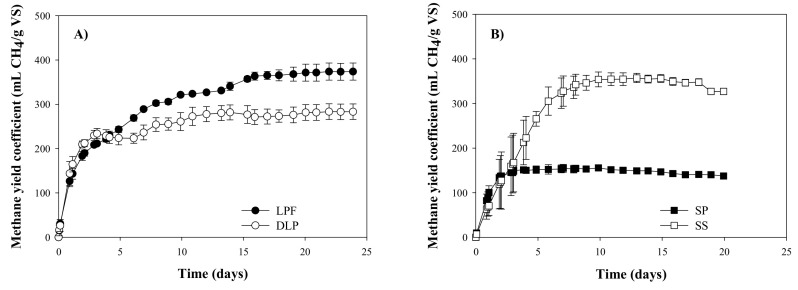
Accumulated methane production for (**A**) LPF and DLP and (**B**) SP and SS expressed as mL CH_4_/g VS against time.

The methane production was monitored throughout the experimental time for each substrate. The methane production was exhausted after 20–25 days in all the cases (Figure 1). The small standard deviations between the replicates for each substrate indicate a good reproducibility of the experiments. The higher standard deviation detected for the SS from days 1 to 5 would be a consequence of differences in the degradation rate, although the degradation was homogeneous after this time (Figure 1). It is worth to note that the methane production starter immediately after the beginning of the experiments, without lag-phases, allowing the use of a first-order model for the kinetic evaluation of the processes. The absence of a lag-phase would indicate that the hydrolysis was not the rate-limiting step [10]. These results were different than the obtained by Serrano et al. [6], which reported a sigmoidal curve in the methane production of the solid phase obtained from a hydrothermal treatment at 65 °C of OMSW. That would indicate that the thermo-malaxation process at 60 °C maintain enough biodegradable compounds in the SP avoiding the lag-phase.

Table 2 summaries the parameters obtained from the application of a first-order model to the experimental data showed in Figure 1A,B, as well as the values of R^2^ and Error (%) to evaluate the goodness-of-fit. The values of R^2^ were higher than 0.95 in all the cases, whereas the Error was lower than 10% except for SS, indicating an acceptable fitting (Table 2). The highest G_max_ value was obtained for LPF, i.e., 358 ± 6 mL CH_4_/g VS, around 34% higher than the DLP, i.e., 267 ± 4 mL CH_4_/g VS (Table 2). This difference could be explained because some of the compounds retained during the phenolic extraction process were susceptible to be biomethanized, reducing the percentage of biodegradable compounds in DLP respect LPF. Phenolic compounds have been reported to be biodegradable at moderate concentrations, although they could be inhibitory at certain concentrations depending on the specific phenolic compounds [11]. The lower methane yield coefficient would be also in line with the lower VS/TS ratio of DLP respect LPF (Table 1). SS presented the highest value of G_max_, i.e., 366 ± 7 mL CH_4_/g VS, slightly higher than the one obtained for LPF (Table 2). Both phases presented a similar concentration of soluble sugars, i.e., around 24 g/kg, which was several times higher than the one determined in DLP and SP (Table 1). The high methane yield coefficient of SS would also be a consequence of the long storage period, i.e., 2 months (Figure 1), which has caused the partial degradation of the compounds and facilitates their subsequent biomethanization. Lü et al. [12] reported that the storage of food waste during several days enhanced the hydrolysis and acidification during a subsequent methanization. The lower G_max_ value of SP would be related to the lower ratio between the sCOD and the VS in the substrate with respect to the other phases, i.e., SS, LPF and DLP (Table 2). These results are in line with Cubero-Cardoso et al. (2020) [13]. These authors reported that the solid phase obtained after the steam-explosion treatment (220 °C and 5 min) of strawberry extrudate produced a methane yield coefficient around 50% lower than the liquid phase.

The values of K′ and R_max_ presented an opposite trend to the one described for G_max_, i.e., DLP and SP presented higher values than LPF and SS (Table 2). This difference would be explained by the presence of compounds in SP and LPF, such as the phenolic compounds, which are susceptible to be biomethanized but that required more time than other compounds presented in SP and DLP. The presence of compounds with very different biodegradation rates would explain the stepwise increase of the methane yield coefficient of LPF shown in Figure 1A.

#### 2.2.2. Effluent Characterisation

The effluents of the biochemical methane potential tests were analysed for evaluating the stability and organic matter degradation after the anaerobic digestion process (Table 3). As it can be seen, the pH values were within the optimal range for the methanogenic activity, ranged between 6.8 to 7.8 [14], indicating that no acidification occurred. The high concentrations of alkalinity at the end of the experimental time also corroborate that the buffering capacity of the system was enough to mitigate the decrease of the pH due to the possible accumulation of acids during the anaerobic degradation of the substrates. The low concentration of sCOD at the end of the experimental time (Table 3) would also corroborate that the hydrolysed compounds were effectively converted into methane instead of being accumulated in the effluents due to an inhibition process by acidification. The acidification can be considered as one of the main issues in the anaerobic digestion of OMSW due to its rapid degradation. For example, Serrano et al. (2019) [15] reported a strong consumption of alkalinity during the anaerobic digestion of OMSW at semi-continuous operation mode, which resulted in a drop of the pH, a rapid increase in the volatile fatty acid concentration, and the final fail of the methane production.

The percentual biodegradability after the anaerobic digestion markedly varied depending on the substrate from 22 to 68% (Table 3). The highest value corresponded to the LPF, which was in line with the high methane yield coefficient described in the previous section. On the opposite, the lowest value corresponded to the SP, i.e., 22% (Table 3). The biodegradability of SP is much lower than the values described for solid phases obtained after thermal treatments of OMSW, which reached values around 55% [6,7]. The difference could be due to use of a three-phases centrifugation process in the present research work, which allow a better separation of the phases and, hence, it entails a higher displacement of soluble and easily biodegradable compounds to the liquid phases compared to a two-phases centrifugation process.

### 2.3. Potential Energy and Valuable Compounds Recovery

**Figure 2:**
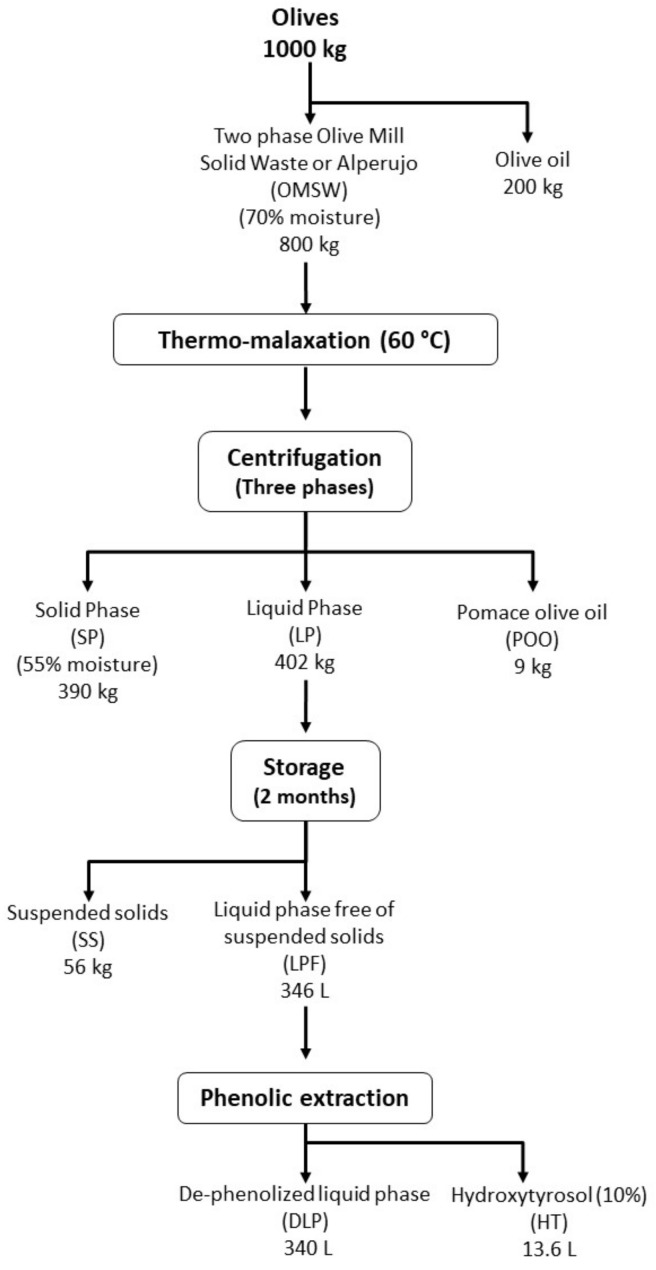
Process scheme (developed by the authors).

The integral valorisation of alperujo can be reached in different ways, depending on the processes to be implemented for the recovery of valuable compounds and the final stabilization of the organic matter. According to the proposed biorefinery process, around 200 kg of extra virgin olive oil can be obtained per ton of olives (Figure 2), which at a market price of €2.568 per kg [16] resulted in a total value of €513.60 per ton of olives. Subsequently, the implementation of a thermo-malaxation at 60 °C and 3-phases centrifugation of the alperujo resulted in the obtaining of 9 kg of pomace olive oil, which has a total market value of €17.96 per ton of olives (€1.995 per kg of pomace olive oil) [16]. The phenolic compound extraction allowed the recovery of 13.6 L of an extract with 10% of hydroxytyrosol (Figure 2). This extract has a very high market price, i.e., €520/L [17], due to its wide range of applications in pharmacy, cosmetics or as food additive [18], although lower prices might be expected if produced at larger scale. The total potential economic value of this phenolic fraction would reach a value of up to €7000 per ton of olives, making it the most valuable resource to be obtained from the proposed biorefinery.

According to the present research, the phenolic compound extraction also reduced the potential energy production due to the lower methane yield coefficient of DLP respect LFP (Figure 1 and Table 2). Concretely, the total methane production would decrease from 35.4 m^3^ of CH_4_/ton of olives to 26.9 m^3^ of CH_4_/ton of olives due to the phenolic extraction process, i.e., around 24% less. Despite of the significant reduction in the methane production, it results in a low impact on the economic feasibility of the biorefinery due the low economic value of the methane, e.g., a similar natural gas has a market price in Europe ranging between €20–32/MWh [19]. This market value is minimal in comparison with the economic interest derived of the recovery of the hydroxytyrosol, hence, making acceptable the above-mentioned reduction of the methane yield coefficient. Despite of the economic value of the generated methane, it is worth mentioning that the anaerobic digestion of the different phases obtained from the biorefinery process also contributes to the stabilization of the organic matter, which is a requirement for an environmentally sustainable process.

## 3. Materials and Methods

### 3.1. Olive Mill Solid Waste Obtained from a Two-Phase Olive Oil Extraction System or Alperujo

Olive mil solid waste obtained from a two-phase olive oil extraction system or alperujo was obtained from the olive mill S.C.A. San Isidro Labrador (Marchena, Spain).

### 3.2. Process Scheme of the Biorefinery Approach

The olive mill solid waste obtained from the two-phase olive oil extraction system or alperujo was thermally treated by thermo-malaxation [20]. The thermo-malaxation consisted in stirring the alperujo slowly at 60 °C for 90 min. After heating and stirring, the alperujo was centrifuged in a three phases decanter, producing a solid phase (SP), a liquid phase (LP), and pomace olive oil (POO) (Figure 2). The whole process was carried out in the facilities of the Instituto de la Grasa (CSIC, Sevilla, Spain) using Pieralisis equipment (Pieralisis, Jesi, Italy) with a capacity of milling of 5000 kg/h. The LP was stored at room temperature in a 1000 L tank for a period of two months. This time helped to settle a suspended solids (SS) phase and to hydrolyze HT precursors in LP, and subsequently increasing the HT in free form. After these two months, a liquid phase free of suspended solids (LFP) was collected and used as a liquid source of phenolic compounds in a subsequent solid-liquid extraction system. LFP was passed through an adsorption column where phenolics were retained. The adsorption material used is under patent preparation but it shows similar phenolic retention capacity that the XAD16 adsorbent resin [21]. Phenolic compounds were removed from the column by a similar volume of ethanol and water. The extracted phenols, mainly HT, were concentrated in a pilot vacuum evaporator until ethanol was completely removed. Once the extraction process was carried out a de-phenolized liquid phase (DLP) was obtained. Samples of SP, SS, LFP, and DLP were collected and stored at −20 °C before the use in the experiments.

### 3.3. Anaerobic Digestion Experimental Procedure

The biogas potential of SP, SS, LFP and DLP phases were evaluated by biochemical methane potential test. In the cases of SP and SS an inoculum/substrate ratio of 2:1 based on VS was used, while in the case of DLP as inoculum/substrate ratio of 2:1 based on CODs was used. Inoculum was collected from the anaerobic digester of the “EDAR COPERO” wastewater treatment plant (Seville, Spain). The inoculum characteristics were pH = 7.8 ± 0.1; alkalinity = 8223 ± 153 mg CaCO_3_/L; TS = 33,195 ± 815 mg/L; VS = 19,875 ± 550 mg/L. The biochemical methane potential test was carried out under the same conditions as described in Trujillo-Reyes et al. [22]. The biochemical methane potential tests were achieved in the time interval required (c.a. 20-day period) to exhaust methane production. All experiments were performed in triplicate. All data are expressed as the mean. ± standard deviation (SD). Comparisons amongst samples were made using one-way analysis of variance (ANOVA) and the Tukey test. A *p*-value < 0.05 was considered significant.

### 3.4. Kinetic Study

Kinetic parameters for the anaerobic processes were determined from the experimental data obtained by a non-linear regression using the software SigmaPlot (version 11.0, SYSTAT Software Chicago, IL, USA). The kinetic model used was the first-order model (Equation (1)), which has been previously applied by other authors [23,24] using the following expression:

G(t) = G_max_ × (1 − e^(−K′ × t)) (1)

where G(t) (mL CH_4_/g VS) is the methane production over time; t is the independent variable, time (d); G_max_ (mL CH_4_/g VS) is the maximum methane production, and K′ is the kinetic parameter (d^−1^). Additionally, r^2^ and error (%) were determined to evaluate the fit and precision of the results previously described by Trujillo-Reyes et al. [22].

R_max_ is the maximum methane production rate (mL CH_4_/(g VS·d) calculated as the following expression:

R_max_ = G_max_ × K′ (2)

### 3.5. Chemical Analyses

The following chemical analyses were used for the characterization of the different phases, for the inoculum, as well as for the effluents from each biochemical methane potential test at the end of the process. The determination of pH, alkalinity, the concentration of total solids (TS), mineral solids (MS) and volatile solids (VS), and soluble chemical oxygen demand (COD) were developed following the recommendations of the Standard Methods of APHA [25]. For determining water-soluble total sugars the anthrone colorimetric method was used [26,27] as previously described [22]. For the determination of total and individual phenolic compounds of SP and SS, a double extraction with an 80% methanol/water solution was applied. A colorimetric method known as the Folin–Ciocalteau method was used for the determination of total phenolic compounds [28].

## 4. Conclusions

The side streams produced showed different biogas potential. The suspended solids and dephenolized liquid phases showed a high methane potential of 366 ± 7 mL CH_4_/g VS and 358 ± 6 mL CH_4_/g VS, respectively, but the solid phase showed a methane yield of only 150 ± 1 mL CH_4_/g VS. The compounds retained during the phenolic extraction process were susceptible to be biomethanized, reducing the percentage of biodegradable compounds in DLP respect LPF. The low concentration of sCOD at the end of all biogas potential tests indicates that the hydrolysed compounds were effectively converted into methane instead of being accumulated in the effluents due to an inhibition process. The economic benefit is mainly derived from the recovery of the hydroxytyrosol, hence, the above-mentioned reduction in the methane yield coefficient is acceptable.

## Figures and Tables

**Table 1:** Physicochemical characterization of the different phases obtained.

		LPF	DLP	SP	SS
TS	mg/L	83,790 ± 470	10,740 ± 220	436,520 ± 7565	235,200 ± 2100
MS	mg/L	13,050 ± 220	2925 ± 95	20,685 ± 625 ^a^	22,085 ± 490 ^a^
VS	mg/L	70,735 ± 425	7815 ± 310	415,835 ± 6 995	213,120 ± 2340
VS/TS		0.84 ± 0.01	0.73 ± 0.03	0.95 ± 0.02	0.91 ± 0.01
sCOD	mg O_2_/L	111,791 ± 899 ^a^	10,868 ± 130	61,653 ± 518	100,938 ± 259 ^a^
pH		4.9 ± 0.1 ^a^	7.3 ± 0.1	4.6 ± 0.1 ^a^	4.9 ± 0.1 ^a^
Total sugars	mg/L	23,224 ± 825 ^a^	725 ± 32	5449 ± 353	24,204 ± 438 ^a^
Total phenols	%	0.39 ± 0.03 ^a^	0.03 ± 0.00	0.20 ± 0.01a	0.73 ± 0.08

^a^ Means signed by letter a are not significantly different *p* < 0.05.

**Table 2:** Kinetics parameters obtained from the different biochemical methane potential tests.

	G_max_ (mL CH_4_/g VS)	R_max_ (mL CH_4_/(g VS·d))	K′ (d^−1^)	R^2^	Error (%)
LPF	358 ± 6	100	0.279 ± 0.018	0.9668	−4.239
DLP	267 ± 4	190	0.711 ± 0.059	0.9576	−5.676
SP	150 ± 1	158	1.056 ± 0.052	0.9808	9.002
SS	366 ± 7	90	0.245 ± 0.015	0.9754	11.888

**Table 3:** pH, Alkalinity, TS, MS, VS, Biodegradability and soluble COD in the biochemical methane potential tests at the end of the experimental time.

		LPF	DLP	SP	SS
pH		7.10 ± 0.02	7.16 ± 0.02	7.37 ± 0.08	7.58 ± 0.06
Alkalinity	mg CaCO_3_/L	5523 ± 52	6200 ± 247	5819 ± 57	6035 ± 271
TS	g/L	20.8 ± 0.2	20.2 ± 0.6	17.7 ± 0.3	18.2 ± 0.2
MS	g/L	9.3 ± 0.1	10.1 ± 0.1	8.5 ± 0.2	8.4 ± 0.1
VS	g/L	11.5 ± 0.1	10.2 ± 0.8	9.1 ± 0.4	9.8 ± 0.1
Biodegradability	%	68	46	22	42
sCOD	mg O_2_/L	1045 ± 20	855 ± 10	480 ± 5	885 ± 5
